# An Analysis of Electrical Impedance Measurements Applied for Plant N Status Estimation in Lettuce (*Lactuca sativa*)

**DOI:** 10.3390/s140711492

**Published:** 2014-06-27

**Authors:** Rafael F. Muñoz-Huerta, Antonio de J. Ortiz-Melendez, Ramon G. Guevara-Gonzalez, Irineo Torres-Pacheco, Gilberto Herrera-Ruiz, Luis M. Contreras-Medina, Juan Prado-Olivarez, Rosalia V. Ocampo-Velazquez

**Affiliations:** 1 Ingeniería de Biosistemas CA, División de Estudios de Posgrado, Facultad de Ingeniería, Universidad Autónoma de Querétaro, Cerro de las Campanas S/N, 76140 Querétaro, Qro., Mexico; E-Mails: antoniom85@hotmail.com (A.J.O.-M.); ramon.guevara@uaq.mx (R.G.G.-G.); irineo.torres@uaq.mx (I.T.-P.); gherrera@uaq.mx (G.H.-R.); mcontreras@hspdigital.org (L.M.C.-M.); 2 Departamento de Ingeniería Electrónica, Instituto Tecnológico de Celaya, Av. Tecnológico y García Cubas S/N, 38010 Celaya, Gto., Mexico; E-Mail: juan.prado@itcelaya.edu.mx

**Keywords:** electrical impedance, characteristic frequency, plant N status, *Lactuca sativa*, plant nutrition

## Abstract

Nitrogen plays a key role in crop yields. Hence, farmers may apply excessive N fertilizers to crop fields, inducing environmental pollution. Crop N monitoring methods have been developed to improve N fertilizer management, most of them based on leaf or canopy optical-property measurements. However, sensitivity to environmental interference remains an important drawback. Electrical impedance has been applied to determine the physiological and nutritional status of plant tissue, but no studies related to plant-N contents are reported. The objective of this article is to analyze how the electrical impedance response of plants is affected by their N status. Four sets of lettuce (*Lactuca sativa* L.) with a different N-source concentrations per set were used. Total nitrogen and electrical impedance spectra (in a 1 to 100 kHz frequency range) were measured five times per set, three times every other day. Minimum phase angles of impedance spectra were detected and analyzed, together with the frequency value in which they occurred, and their magnitude at that frequency. High and positive correlation was observed between plant N content and frequency values at minimum phase angle with no significant variations detected between days of measurement. These results suggest that electrical impedance can be sensitive to plant N status.

## 1. Introduction

Nitrogen (N) is a plant's most-required mineral nutrient due to its importance in several plant cell components; its concentration in plant tissue is the highest of all mineral nutrients [1]. It has a notable effects on plant photosynthesis, leaf respiration, and crop productivity [2]. Usually, soil N supply is limited, and improved N-fertilizer-management according to crop N requirements is needed to optimize plant yields [3]. Thus, it is essential for farmers to be informed regarding crop and soil N status to make better decisions about N fertilizer application [4]. Nitrogen overfertilization causes environmental pollution and increases production costs. Runoffs move N excess in the soil to surface water bodies, where it produces the eutrophication phenomenon; N-excess leaching pollutes groundwaters, and nitrogenous oxides (e.g., NO, N_2_O, NO_2_) released into the atmosphere by overfertilized-N crops encourage global warming [5]. In addition, leafy vegetables (e.g., lettuce, cabbage, spinach, *etc.*) become harmful for human consumption when fertilized with high amounts of nitrate as it may cause diseases such as methaemoglobinaemia, and gastric and bladder cancer [6].

Recently, researchers have focused on improving N fertilizer management in crops developing new crop-N-status measuring methods. Non-invasive methods involving leaf or canopy reflectance properties have been mostly studied and applied to determine crop N status [2]. Due to their low-cost, hand-held chlorophyll meters (e.g., SPAD) have been utilized to estimate plant N status [7]. The Dualex hand-held meter measures polyphenolics—which are carbon-based compounds also used as leaf N indicators—by means of leaf chlorophyll fluorescence [8]. A chlorophyll/polyphenolics ratio calculated using SPAD and Dualex measurements has been proposed as a better plant N status estimator [9,10]. Canopy-level sensors are capable of measuring crop N status in larger areas by analyzing NIR, VIS, and UV reflectance spectra [11]. In addition, digital image processing algorithms have been developed to analyze hyper-spectral data, fluorescence, and NIR-, VIS- and UV imagery [12] detected via digital cameras [4,13]. The normalized differential vegetation index (NDVI) calculated using NIR canopy reflectance has been studied to determine crop N requirements [9]. Hyper-spectral imaging systems have been developed to assess wheat N content [3], and detect N-overfertilized fields [14]. Lately, satellite-mounted hyper-spectral sensors have been developed and studied to detect nutritional status in crop fields [15,16]. In addition, according to Thoren and collaborators [17], plant N status can be correlated with laser-induced chlorophyll fluorescence.

Non-invasive methods have limitations as to their environmental sensitivity and confounding factors (*i.e.*, soil condition, light intensity, canopy shape and color, *etc.*) [12]. Plant-tissue electrical properties are also modified by their physical structure, the chemical processes within them, or the combination of both [18]. According to its response to electrical fields, biological tissue may be considered as a volume conductor (due to specific conductivities) or a dielectric (due to relative permittivities), and ions therein as charge carriers [19]. Generally, a cell membrane can be represented as a capacitor because it is regarded as a dielectric, whereas intracellular and extracellular fluids can simulate a resistor [20]. Electrical energy flowing through biological tissue (or any material) by means of charge carriers can be dissipated by those resistors, and stored by capacitors. Electrical impedance is a passive electrical property that determines how an applied alternating-current flow generated by an external electrical field is “impeded” by biological tissues (or any other materials) [20]. A combination of resistive and capacitive effects contributes to alternating-current flow opposition, which is related to the impedance magnitude (mainly, due to resistive effects) and the impedance phase (mainly, due to capacitive effects) of electrical impedance.

Several researchers have reported methodologies based on electrical impedance measurements to determine the physiological status of biological tissues. Numerous articles report medical applications of electrical impedance measurements such as the analysis of electrical impedance responses given by various types of human body tissues, as studied by Miklavčič and collaborators [21]. In addition, Meeuwsen *et al.* [22] applied bioelectrical impedance to determine body mass index and body fat in adult people, whereas Rösler *et al.* [23] assessed nutritional and hydration status in elderly people. Also, electrical impedance measurements have been studied to determine plant physiological status. Zhang and Willison [24] analyzed potato tubers and carrot roots using a double-shell model. A Cole-Cole model was applied to analyze the electrical impedance response of Scots pine needles [25]. Jackson and Harker [26] detected damaged tissue in bruised apples; Liu [27] determined a relationship between electrical impedance and fruit biochemical properties (pH, sugar content, ripening). A willow root system was also assessed via electrical impedance measurements [28]; a portable system was developed by He and collaborators [29] to detect plant water status in tomato; and Borges *et al.* [20] detected hydric stress and diseases in three forest tree species. Few studies however have focused on detecting nutrition status in plants. Greenham and collaborators [30] studied electrical impedance measurements taken from phosphorous- and potassium deficient *Trifolium subterraneum* plants, and Tomckiewicz and Piskier [12] assessed tomato plant stress caused by lack of nutrients.

As seen, electrical impedance has been widely used to determine the physiological status of biological tissues. Similarly, plant physiological status and agricultural product quality have been analyzed using electrical impedance measurements due to their simplicity and effectiveness. Furthermore, electrical impedance measurements are less sensitive to environmental variables and other factors than non-invasive methods used to determine crop physiological status. Despite this, there is little research concerning plant nutrition status detection, and there are no studies focused on analyzing plant N status effect on electrical impedance measurement values. Therefore this article focuses on the analysis of how the electrical impedance response of plants is affected by their N status. It is described a novel methodology based on the detection of the minimum phase angle in a measured electrical impedance spectum, and the correlation between the frequency at which it occurred and plant total N. As mentioned above, monitoring N status is specifically important in leafy crops because they become harmful for human consumption when N overfertilized. Hence, the cultivar PVP8900099 of lettuce (*Lactuca sativa* L.) was selected due to its short crop cycle and because it is recommended for growing in floating bed systems.

## 2. Experimental Section

### 2.1. Experimental Site

Lettuce was grown inside a greenhouse with a covered area of 108 m^2^ (9 m wide × 12 m long), and 5 m height. The greenhouse was built in a double-layer chapel-type structure made with 5.08-cm PTR, and covered with 0.2 mm-thick polyethylene. The greenhouse is north-south oriented, with roof and lateral ventilation covered with an anti-aphid mesh. This experimental site is located at Universidad Autónoma de Querétaro Campus Amazcala, in Queretaro, Mexico, at the coordinates 100°16′W, 20°42′N, and at 1920 m altitude. Average room temperature ranged from 22 to 24°C.

### 2.2. Nutrient Solutions

Four nutrient solutions (NS) were employed, each with different amounts of nitrate (as a nitrogen source). Solutions were made according to the Steiner nutrient solution [31]. Table 1 shows the chemical composition of Steiner nutrient solution corresponding to each nitrogen treatment: T100 as standard Steiner solution, T75 as Steiner solution with 25% less nitrate, T50 as Steiner solution with 50% less nitrate, and T25 with 75% less nitrate.

### 2.3. Lettuce Cultivation

A randomized complete block design was used in this experimental setup. Two hundred lettuce seeds were used in this experiment. Lettuce seedlings were grown in 128-cell styrofoam trays, which were previously washed and subsequently disinfected with quaternary salts (10 mL/L). Three quarters of each cell was filled with wet peat moss; then, seeds were placed between 0.5–1 cm deep; finally, the top quarter was filled with vermiculite. The trays were covered with dark plastic sheets and placed in a dark room at a higher temperature in order to encourage seed germination. Prior to transplantation, the trays were put in the greenhouse after reaching germination (three-to-five days following planting). Then, plant seedlings (with 3-4 true leaves) were transplanted into a floating bed system 18 days after planting. Four 1-m^2^, 10-cm deep floating beds (one per treatment) with a density of 30 plants/m^2^ were used and filled with nutrient solution according to its corresponding treatment. The solutions were stirred for 5 min. every morning per floating bed in order to maintain adequate oxygen levels in roots. Due to this lettuce cultivar has a short crop cycle, nutrient solution composition was not varied, but every week floating beds were refilled with water and its corresponding NS.

### 2.4. Measurement Procedure

**Figure 1.**
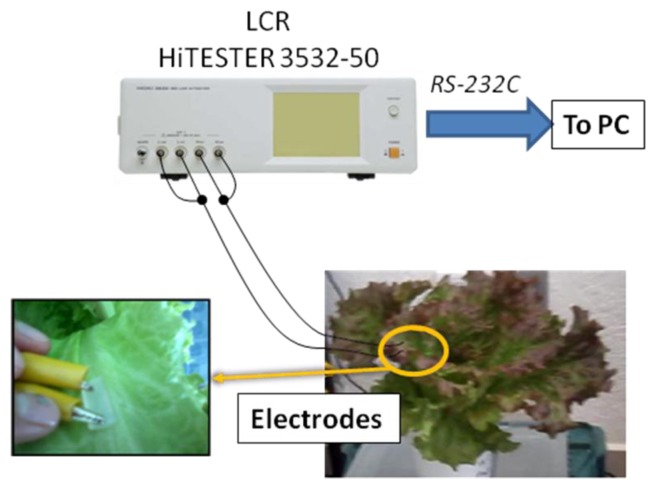
Electrical impedance measurement in lettuce.

Electrical impedance measurements were carried out 45 (day 1), 47 (day 2), and 49 (day 3) days after planting (following a NS refilling procedure at day 44 after planting, when lettuce was fully developed) in order to monitor the plant N- and the electrical impedance variations during the elapsed time between each NS-refilling procedure. Measurements were taken between noon and 3 p.m when plant photosynthetic activity is high and N concentration is stable. Five plants were measured per floating bed per day. Measurements were taken by inserting two stainless steel needle electrodes into a young-leaf midrib, at a point closest to the stem. The electrodes were removed from measured plant, washed with distilled water, and reinserted into the next plant when the electrodes dried. The electrodes were 0.75 cm long, and 1.5 cm apart from each other (Figure 1). Electrical impedance was measured by using a LCR HiTESTER 3532-50 (Hioki E. E. Corporation, Nagano, Japan). Before measurements, the LCR-meter was open- and short-circuit calibrated, as suggested by the instruction manual [32]. In order to avoid electrode polarization phenomenon, frequencies above 1 kHz were used [12]. The device was set to make a frequency sweep from 1 to 100 kHz with 1 kHz steps, and applying 50 μA of constant rms current. The data was sent to a PC through a RS-232C serial port.

After measuring their electrical impedance, plants tested were classified according to treatment and measurement date, and then they were sent to a laboratory in order to measure total nitrogen content according to the Kjeldahl method.

### 2.5. Data Analysis And Minimum Impedance Phase Criterion

**Figure 2.**
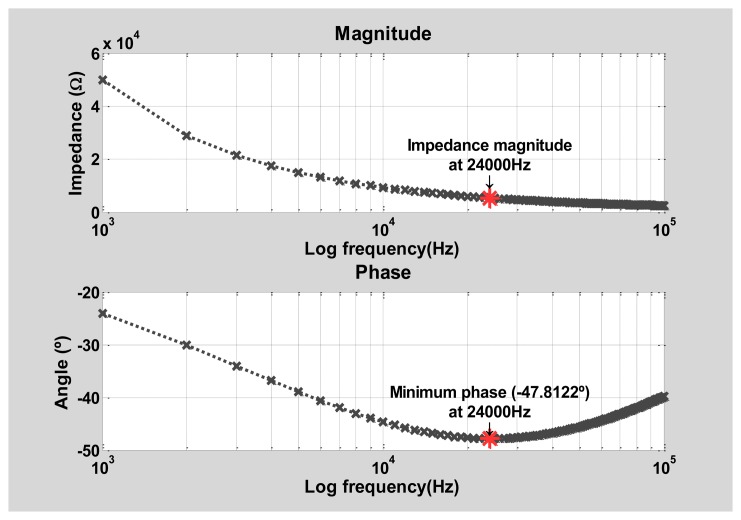
An example of an electrical impedance spectrum obtained. This measurement was taken on day 1 from a lettuce in T50-treatment.

Averages of N-content measurements (five per treatment) were plotted against treatments per day. Nitrogen content data was analyzed using one-way ANOVA per treatment in order to determine significant variations between days, and plant N-content correlation against treatments was estimated using the Pearson coefficient. Plant electrical impedance spectra were analyzed using MATLAB software. An example of a spectrum obtained is shown in Figure 2. This behavior was observed in all the measured spectra; while magnitude values decayed monotonically as frequency increased, phase values had a parabolic-like behavior with a minimum value at certain frequency. Those minimum phase angle (*φ*_min_) were detected in the electrical impedance spectra and analyzed, as well as the frequency values in which they occur (*f*_min_) and the magnitude value at *f*_min_ (*Z*_min_). Five measurements of *φ*_min_, *f*_min_, and *Z*_min_ were averaged per treatment and plotted against their corresponding average N-content value at the same treatment on the same day. Also, one-way ANOVA was applied to *φ*_min_, *f*_min_, and *Z*_min_ data per treatment, and their correlation against plant N-content was determined by calculating the Pearson coefficient.

## 3. Results and Discussion

### 3.1. Lettuce Total Nitrogen

**Figure 3.**
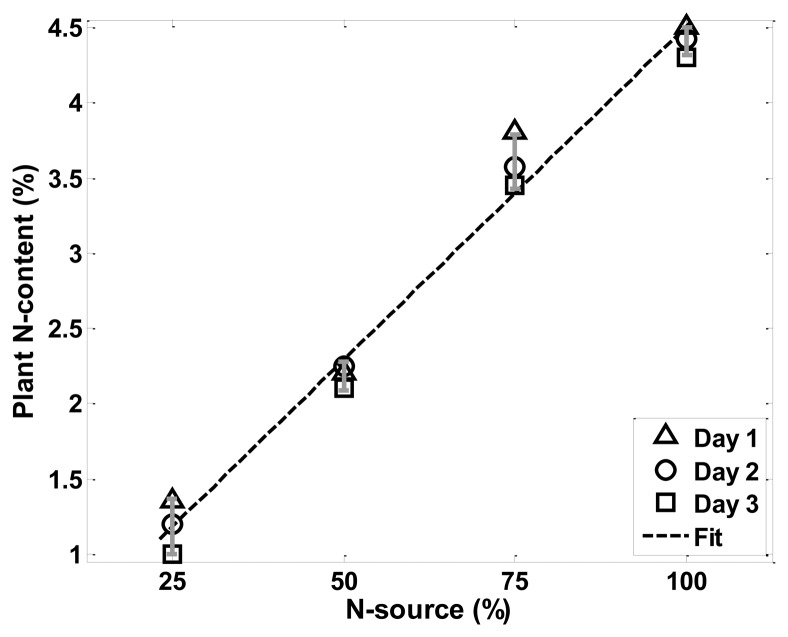
N-content averages (n = 5) related to the percentaje of nitrogen supplied by using standard Steiner solution per treatment per day. The dashed line belongs to the linear fitting function (R^2^ = 0.997).

In order to demonstrate N-content variations in lettuce according to N-source concentration, plant total-N was measured and analyzed. The Pearson coefficient was calculated to assess the correlation between nitrogen content and treatments. High coefficient values were obtained per day as shown in Table 2. The plant N-content showed a linear and directly proportional behavior between treatments per day (Figure 3). N-content variability between days was analyzed using a one-way ANOVA. Nevertheless, ANOVA p-values suggested no significant N-content differences between days (p > 0.05) at each treatment (Table 3). These results suggest that lettuce did not react significantly to N deficiency between each NS refilling.

Due to the high correlation and lack of significant differences between days (according to one-way ANOVA), a linear function was generated by means of linear least squares using three-day data. Equation (1). describes the relationship between N source (as a percentage of total N supplied by standard Steiner solution) and plant total N-content (%):

(1) TotalN=0.0444*Nsource+0.07083

### 3.2. Relationships between Minimum Impedance Phase Variables and N-content

**Figure 4.**
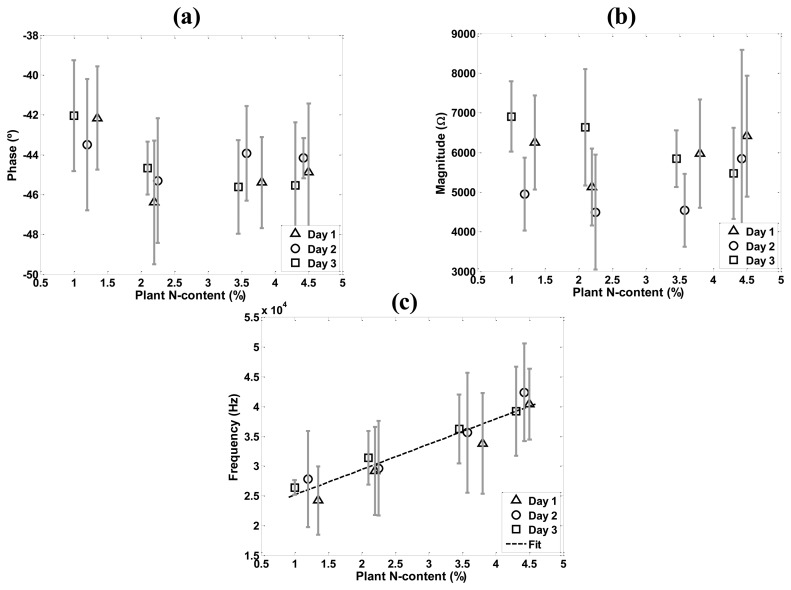
Relationships between each of *φ*_min_, *Z*_min_, and *f*_min_ and N-content per day: (**a**) N-content *vs. φ*_min_, (**b**) N-content *vs. Z*_min_, and (**c**) N-content *vs. f*_min_. To relate N-content and *f*_min_ a fitting function was generated using averaged data (n = 5) per treatment (R^2^ = 0.99). The bars indicate standard deviations (±σ).

The correlation between nitrogen content and each of *φ*_min_, *Z*_min_, and *f*_min_ was assessed and analyzed. Figure 4 displays the relationship between N-content and *φ*_min_ (Figure 4a), *Z*_min_ (Figure 4b), and *f*_min_ (Figure 4c). A one-way ANOVA applied per treatment (Table 4) shows no significant changes between days in *φ*_min_ and *f*_min_ (p < 0.05), but *Z*_min_ seems to have a significant variation at T25 (p < 0.05). However, there is low correlation between *Z*_min_ and plant N-content as seen in Figure 4b. Pearson coefficients were calculated to assess the correlations between nitrogen content and each of *φ*_min_, *Z*_min_, and *f*_min_. As shown in Table 5, correlation coefficients are low between N-content and both *φ*_min_ and *Z*_min_ on day 1 and day 2, but on day 3 all the three variables have high values: *φ*_min_ and *Z*_min_ are negatively correlated, while *f*_min_ is positively correlated to N-content.

The behavior of measurements displayed in Figure 4 are confirmed by The Pearson coefficient values showed in Table 5. Figure 4a,b show no correlation between N-content and *φ*_min_ and *Z*_min_ respectively. In addition, *Z*_min_ data show high and non-monotonic variation between days. However, according to Table 3b regarding the relationship between *f*_min_ and plant N-content, Pearson coefficients were high and positive not only on day 3, but all three days. This high and positive coefficient values are clearly related to data displayed in Figure 4c. Due to this N -content *vs. f*_min_ relationship behavior, a fitting linear function was generated (dashed line in Figure 4c). Equation (2) relates plant N-content (%) and *f*_min_ (Hz):

(2) $$f_{\min}=4262.121\times \text{Ncontent}+20887.38$$

The frequency at the minimum of the impedance phase angle—or at the maximum of the impedance spectrum in the complex plane—is also called characteristic frequency [19]. A cell membrane behaves as a short circuit when the characteristic frequency is reached [27]. Although electrode polarization phenomenon is negligible in the frequency range from 1.5 to 20 kHz [12], the results of this study show that characteristic frequencies of leaf midrib above that range are more sensitive to plant total-N. Methodologies similar to the one proposed in this article have been developed. A peak of impedance phase angle (θ = −32.7°) was used to detect the characteristic frequency (1 kHz) of maize roots to assess the mycorrhizal colonization impact of electrical impedance and capacitance [33]. A bigger plant root surface or the enhanced absorption area by fungal hyphae could cause those changes. However, although Cseresnyés *et al.* [33] found a linear correlation between electrical impedance and root system size, results shown in this study demonstrate a low correlation between plant total-N and electrical impedance magnitude at characteristic frequencies. The ripening process in apple and tomato decreases characteristic frequency [27]. Frequencies of 1, 5, 50, 100, 200 to 500 kHz are generally used to determine total human body water content [27]. In plant tissue, two characteristic frequencies are found in the complex plane of impedance spectrum of Scots pine needles and stems under frost hardening [34, 35]. Repo *et al.* [34] found that the higher characteristic frequency increased monotonically in stems (from 410 Hz to 49.7 kHz) as frost damage rose due to a decrease in membrane capacitance and intracellular resistance. However, the characteristic frequency of leaf midribs rises according to plant total-N. This could be due to high quantities of nitrate ion, which cause extracellular resistance to decrease, relaxation time to fall, and characteristic frequency to rise. In addition, characteristic frequency falls as dry matter content increases and water content decreases [35]. Low total-N in plant tissue causes a dry matter content decrement, which means less water contents and low nitrate ion concentration.

## 4. Conclusions

According to the experimental setup developed in this study, results suggest N-content was highly and positively correlated with the N source concentration, but there had no significant differences between days. A linear function was generated by linear least-squares to relate plant N-content with the N source concentration. After analyzing the relationship between plant electrical impedance and plant N-content, high and negative correlation with *φ*_min_ and *Z*_min_ was found on day 3. However, N-content had a high and positive correlation with *f*_min_ all the days of measurement. The results suggest characteristic frequencies (frequencies at minimum phase angles) may be used as a plant total-N-content estimator, at least for lettuce. Due to high correlation between N-content and *f*_min_, a linear function was generated by linear least-squares to match this behavior. A novel methodology is proposed in this article to analyze plant electrical impedance measurements based on characteristic frequency detection by measuring minimum phase angles within frequency range from 1 to 100 kHz. Unlike non-invasive methods, electrical impedance measurements are not time consuming, do not require expensive equipment, and they could be used to detect overfertilized plants. Despite the high correlation between N-content and *f*_min_, and the strong influence of nitrogen in several plant physiological processes, the effect of diseases, other nutrients, and physiological variables in electrical impedance measurements should however be assessed before its use for commercial purposes.

## Figures and Tables

**Table 1.** Chemical composition (in mg·L^−1^) of nutrient solutions based on Steiner solution.

	**T100**	**T75**	**T50**	**T25**
Nitrogen (N)	168	127	84	42
Phosphorus (P)	31	31	31	31
Potassium (K)	299	273	273	161
Calcium (Ca)	150	150	150	150
Magnesium (Mg)	48	48	48	48
Sulfur (S)	87	113	94	113
Iron (Fe)	3	3	3	3
Manganese (Mn)	1.97	1.97	1.97	1.97
Boron (B)	0.44	0.44	0.44	0.44
Zinc (Zn)	0.11	0.11	0.11	0.11
Copper (Cu)	0.02	0.02	0.02	0.02

**Table 2.** Statistical analysis of N-content data: Pearson correlation coefficient values.

	**day 1**	**day 2**	**day 3**
Pearson coefficients (ρ)	0.98	0.99	0.99

**Table 3.** Statistical analysis of N-content data: one-way ANOVA between days per treatment.

	**T25**	**T50**	**T75**	**T100**
ANOVA *p*-values	0.13	0.37	0.10	0.10

**Table 4.** Statistical analysis of *φ*_min_, *Z*_min_, and *f*_min_ variable behavior *vs.* N-content: one-way ANOVA *p*-values between days per treatment.

	**T25**	**T50**	**T75**	**T100**
*φ*_min_	0.68	0.59	0.48	0.73
*Z*_min_	0.02	0.06	0.09	0.74
*f*_min_	0.63	0.90	0.92	0.97

**Table 5.** Statistical analysis of *φ*_min_, *Z*_min_, and *f*_min_ variable behavior *vs.* N-content: Pearson coefficient for each of *φ*_min_, *Z*_min_, and *f*_min_ variables *vs.* N-content correlations.

	**N-content**		

	**day 1**	**day 2**	**day 3**
*φ*_min_	−0.46	−0.08	−0.89
*Z*_min_	0.35	0.51	−0.98
*f*_min_	0.97	0.96	0.99
